# Spray Drying of a Subcritical Extract Using *Marrubium vulgare* as a Method of Choice for Obtaining High Quality Powder

**DOI:** 10.3390/pharmaceutics11100523

**Published:** 2019-10-11

**Authors:** Aleksandra Gavarić, Jelena Vladić, Rita Ambrus, Stela Jokić, Piroska Szabó-Révész, Milan Tomić, Marijana Blažić, Senka Vidović

**Affiliations:** 1 Faculty of Technology, University of Novi Sad, Bulevar cara Lazara 1, 21 000 Novi Sad, Serbia; cvejina@uns.ac.rs (A.G.); vladicjelena@gmail.com (J.V.); 2 Institute of Pharmaceutical Technology and Regulatory Affairs, University of Szeged, Eotvos 6, 6720 Szeged, Hungary; arita@pharm.u-szeged.hu (R.A.); revesz@pharm.u-szeged.hu (P.S.-R.); 3 Faculty of Food Technology Osijek, Josip Juraj Strossmayer University of Osijek, Franje Kuhača 20, 31000 Osijek, Croatia; 4 Faculty of Agriculture, University of Novi Sad, Trg Dositeja Obradovica 8, 21 000 Novi Sad, Serbia; milanto@polj.uns.ac.rs; 5 Department of Food Technology, Karlovac University of Applied Sciences, Josip Juraj Strossmayer Square 9, 47000 Karlovac, Croatia; marijana.blazic@vuka.hr

**Keywords:** *Marrubium vulgare*, subcritical water extraction, spray drying, powder characterization

## Abstract

White horehound (*Marrubium vulgare* L.), is a grey-leaved perennial herb, belonging to Lamiaceae family, distributed in Eurasia and northern Africa. Despite the fact that *M. vulgare* has been used since ancient times in treating diverse diseases, it is only in the last decade or so that scientists have been able to lay the foundation for its potential pharmacological actions from the results observed through the prism of ethnopharmacological use of this species. The novelty of this study was that subcritical water extraction, acknowledged as a powerful extraction technology to recover phenolic compounds, was coupled with spray drying. The subcritical horehound extract, obtained using optimal process parameters, was used as a liquid feed in spray drying. Maltodextrin was used as a carrier in a concentration of 10%. Thus, two *M. vulgare* powders, carrier-free and 10% MD, were produced. Comprehensive powders characterization was conducted in order to evaluate their quality. Results confirmed that spray drying can be used as a method of choice for obtaining high quality horehound powders which kept the amorphous structure constant after 6 months.

## 1. Introduction

The aerial parts and root of *Marrubium vulgare* L. have been traditionally used in the Mediterranean areas of Europe and North Africa. More familiar as a white horehound, it belongs to the genus *Marrubium*, formed by nearly 30 species [1]. This genus is an abundant source of secondary metabolites, including nine different types of diterpenes and their derivatives, more than ten flavonoid constituents, phenylpropanoids and theirglycosides [2]. The most studied secondary metabolite, marrubiin, a labdane diterpenoid, was isolated for the first time from *M. vulgare* leaves [3]. The reason why this chemotaxonomic marker was the subject of many studies is due to the fact it possesses several biological activities such as antioxidant, anti-inflammatory and vasorelaxant effects [4]. This perennial plant, with morphological characteristics reminiscent of a mint [5], shows numerous diverse pharmacological effects. It was reported that a hydroalcoholic extract of the aerial parts and root of horehound exerts significant antispasmodic activity, which means that it can be used as an expectorant for acute/chronic bronchitis, colds and in cases of asthma [6]. According to another study, the hydroalcoholic extract also shows analgesic effects when administered per os or intraperitoneally [7]. A series of in vivo experiments were performed in rats seeking antidiabetic effects in horehound. The results were positive, since there was a more than 60% decrease of the blood glucose level when aqueous extracts were administered [8]. The methanol extract of horehound herba revealed moderate activity when evaluated against five Gram positive bacteria: *Bacilus subtilis, Sthapylococcus epidermidis* and *S. aureus*, *Pseudomonas vulgaris* and *Escherichi coli* [9]. Furthermore, this plant demonstrated a strong effect against methicillin-resistant *Staphylococcus aureus*. [10]. Beside its medicinal use, extracts of horehound herba are also used as flavouring agents, especially by the brewing industry as a substitute for hops [5], in candies [11], and as an ingredient of cough pastilles [12].

According to a market research report, horehound was reported as the top-selling herbal dietary supplement ingredient in mainstream US retail outlets for the fifth consecutive year. In this channel, horehound supplement sales in 2017 increased for 12.3% from 2016 [13]. Records of the medicinal use of horehound confirm that this herb is still commonly used for its expectorant and cough-suppressant properties, especially in the form of cough drops and lozenges. According to European Medicines Agency guidelines, horehound is usually used in combination with 3 to 5 herbal substances in Europe. In agreement with guidelines from the US FDA, dietary supplements appear in a number of forms inclusive of tablets, powders, capsules, softgels, gelcaps and liquids. Powders have many benefits over liquid extracts including higher stability, reduced bulk size, higher concentration of bioactives, simple manipulation and shipment, and finally easier standardization [14]. Therefore, spray drying imposes as technique of choice for obtaining a solid phase herbal powders from liquid feed in a single step.

The main purpose of this study was to estimate the efficiency of spray drying technology to microencapsulate phenolic compounds from horehound subcritical extract obtained at optimal conditions of process parameters. This extract, used as liquid feed, was obtained through subcritical water extraction which has been acknowledged as a powerful extraction technology to recover phenolic compounds from different matrices [15,16]. The obtained powders were further investigated with reference to their physical and chemical properties. To the best of our knowledge, the subcritical horehound extract has not been applied yet as a liquid feed for spray drying process.

## 2. Materials and Methods

### 2.1. Plant Material

*M. vulgare* harvested in 2015 was bought from Chamomilla (Banatski Karlovac, Serbia) a local supplier of cultivated plants. The aerial parts of *M. vulgare* were air-dried in a thin layer, collected in the paper bags, and stored at room temperature. Afterwards, the dried *M. vulgare* herba was grounded in a domestic blender and the particle size of grounded material was determined using vibration sieve sets (CISA, Cedaceria, Spain). The mean particle size of *M. vulgare* herba used in investigation was 0.28 mm.

### 2.2. Chemicals

Reagents used in the various methods, 1,1-diphenyl-2-picrylhydrazyl hydrate (DPPH), Folin-Ciocalteu reagent and (±)-catechin were purchased from Sigma (Sigma-Aldrich Chemie GmbH, Sternheim, Germany). The following reagents were also purchased from Sigma-Aldrich Chemie: iron (III)-chloride, potassium hexacyanoferrate (III), sodium hydrogen phosphate anhydrous, sodium dihydrogen phosphate andtrichloroacetic acid. Gallic acid was purchased from Sigma (St. Louis, MO, USA). Maltodextrin of dextrose equivalent (DE) 16.5–19.5 (Sigma-Aldrich Chemie GmbH) was used as a carrier material. All other chemicals and reagents were of analytical grade.

### 2.3. Liquid Extract and Liquid Feed Preparations

Subcritical water extraction (SWE) at optimal process conditions (temperature of 200 °C, extraction time of 20.29 min and absence of HCl) defined in our previous study, was used to obtain liquid extract which was further used as a liquid feed. The scheme of subcritical water extraction apparatus used is presented in previously published paper [17]. In certain amount of liquid feed no carrier was added prior to the drying. Maltodextrin (MD) of dextrose equivalent (DE) 16.5–19.5 was used as a carrier material. Procedure of preparation of liquid feed with 10% MD was descibed in our previous study [14]. Table 1 with composition of prepared formulations, amount of added carrier and drying conditions is provided down below.

### 2.4. Spray Drying Process and its Efficiency

**Figure 1:**
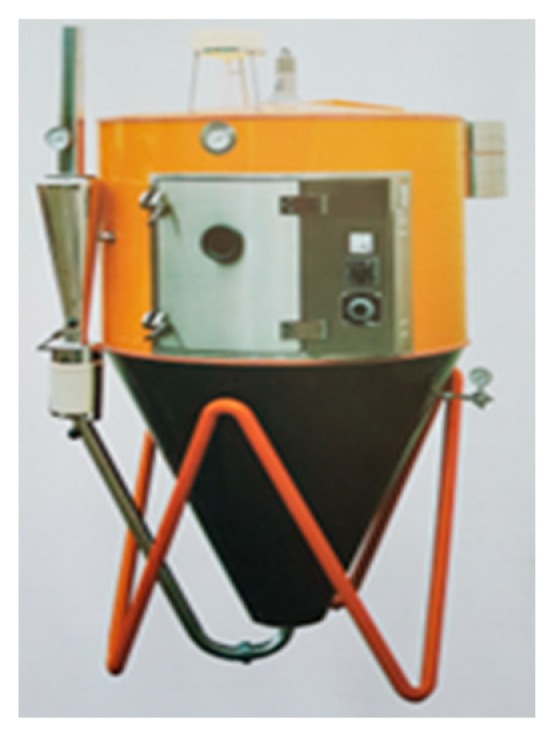
Pilot scale spray dryer.

The pilot scale spray dryer (APV Anhydro AS, Søborg, Denmark) used for spray drying of prepared liquid feed is presented in Figure 1. A laboratory peristaltic pump was used to transfer the liquid feed into the drying chamber. For each run, 2 L of liquid feed was dried. Liquid feeds were dried at inlet temperature, *T*_i_ = 130 ± 5 °C, while outlet temperature, *T*_0_ was maintained at 75–80 °C. During the production of the dry extract (powder), rotary disk, within atomizer, delivered speed from 20,000 to 21,000 rpm. The obtained powder was separated from heating medium in a cyclone and collected in glass bottles, sealed and kept protected from air and humidity. The particle production efficiency (i.e., powder recovery) is determined gravimetrically as ratio of mass of the powder obtained in the collecting vessel after spray drying and mass of total solids measured in the liquid feed. Process efficiency is expressed as the weight percentage.

### 2.5. Analysis of MVPs Stability Properties

#### 2.5.1. Moisture Content

Moisture contents of MVPs were determined according to standard procedure described in the official Pharmacopeia (Ph. Jug. IV). The gravimetric method, based on water removal by heating, was carried out in an oven at 105 °C until achieving constant mass. Measurement of the moisture content was performed promptly after the spray drying. All experiments were performed in three replicates.

#### 2.5.2. Hygroscopicity

All powder samples (approx. 1 g) were placed in desiccator supplied with NaCl saturated solution (70% RH) at 25 °C. The hygroscopicity was measured after 48 h. Hygroscopicity was expressed as a gram of absorbed water per 100 g of dry extract powder. All experiments were performed in three replicates [14].

### 2.6. Analysis of Mvps Solubility and Wettability Properties

#### Water Solubility (WSI) and Water Absorption (WAI) Indexes

The WSI and WAI were determined according to a previously described method [18]. Certain amounts of powder (1.25 g) and water (15 mL) were vigorously mixed in a 50 mL centrifuge tube. Afterwards, the mixture was incubated in a water bath at 30 °C for 30 min, and centrifuged for 15 min at 3000 rpm. The supernatant was decanted in a pre-weighed Petri dish, while particles were concentrated as a solid pellet at the bottom of the centrifuge tube. Both supernatant and pellet were placed in an oven and dried at 105 °C overnight. The amount of solids in the dried supernatant was calculated as a percentage of the total dry solids in the 1.25 g of sample, and represents WSI. WSI, reconstitution property, is used as an indicator of degradation of powder constituents. WAI was calculated as the mass of solid pellets remaining after centrifugation divided by the mass of the original dry sample. WAI is a measure of the products ability to absorb water. WAI depends on the availability of hydrophilic groups and on the gel-forming capacity of macromolecules. The hydrophilic groups are responsible for binding of water molecules. The low WAI indicates better stability during the storage. All experiments were performed in three replicates.

### 2.7. Analysis of Mvps Flow Behavior Properties

#### 2.7.1. Bulk Density

Bulk density was determined by measuring the volume of a known mass of powder sample in a graduated glass cylinder. *M. vulgare* powder (1 g) was placed in a 25 mL graduated cylinder. Afterwards, the bulk density was calculated from the difference between the mass of empty glass cylinder and the mass of glass cylinder with powder sample. Bulk density was expressed as mg of powder per mL.

#### 2.7.2. Powder Characterization

Powder flowability, a key property in filling and by calculating the Hausner ratio and the Carr Index (CI). The Hausner ratio is calculated from the ratio between the bulk and tapped densities of the powder. The Carr Index is another measure of flowability, also calculated from the two densities of the powder [19].

#### 2.7.3. Particle Size Analysis

In order to measure the particle size distribution of the prepared powders, LEICA Image Processing and Analysis System (LEICA Q500MC, LEICA Cambridge Ltd., Cambridge, UK) was used. The size was determined using 350 particles per product. The particles were described in detail by their length, breadth, surface area, perimeter and roundness. The Malvern apparatus (Malvern Mastersizer Scirocco 2000; Malvern Instruments Ltd., Worcestershire, UK) was used for laser diffraction required for determination of powders particle size distributions. The sample (approx. 1 g) was loaded into the feeder tray. The dispersion air pressure was fixed at 2.0 bar to determine if particle attrition has occurred. Obscuration was kept between 10.0% and 15.0% throughout the whole measurement duration. The particle size distribution was characterized by the D (0.1), D (0.5) and D (0.9) values and the specific surface area (SSA).

#### 2.7.4. Morphology-Scanning Electron Microscopy (SEM)

The morphology of the MVPs particles was examined by SEM (Hitachi S4700, Hitachi Scientific Ltd., Tokyo, Japan). In order to induce electric conductivity on the surface of the samples, a sputter coating apparatus (Bio-Rad SC 502, VG Microtech, Uckfield, UK) was applied. The air pressure was 1.3–13.0 mPa.

### 2.8. Analysis of Mvps Crystallographic and Thermal Properties

#### 2.8.1. Differential Scanning Calorimetry Analysis (DSC)

The Mettler Toledo DSC 821e thermal analysis system with the STARe thermal analysis program V6.0 (Mettler Inc., Schwerzenbach, Switzerland) was used for DSC measurements. The sample (approx. 2–5 mg) was examined in the temperature range between 25 °C and 300 °C. The heating rate was 10 °C min^−1^. During the DSC investigation, argon was used as inert carrier gas, at a flow rate of 10 L/h.

#### 2.8.2. X-ray Powder Diffraction Analysis (XRDP)

The physical state of samples was evaluated by X-ray powder diffraction (XRPD). The BRUKER D8 advance X-ray powder diffractometer (Bruker AXS GmbH, Karlsruhe, Germany) with Cu K λI radiation (λ = 1.5406 Å) and a VÅNTEC-1 detector (Bruker AXS GmbH) were used for analyses of diffraction patterns. Scanning of samples were performed at 40 kV and 40 mA. The angular range was 3°–40° 2θ, at increment time of 0.1 s and increment size of 0.007°. All operations, including Kα2 stripping, background removal and smoothing of the area under the diffractograms peaks, were performed using the DIFFRACplus EVA software. The structural characterization by DSC and XRPD analyses were repeated after 6 months using the samples stored at room temperature in exicator.

### 2.9. Analysis of Mvps Bioactive Compounds

#### 2.9.1. Total Phenol Content

The contents of total phenolic compounds (TP) in horehound herbal powders were determined by the Folin–Ciocalteu procedure [20]. Gallic acid was used as standard compound for preparation of calibration curve, and absorbance of the samples was measured at 750 nm (6300 Spectrophotometer, Jenway, Dunmow, UK). Content of phenolic compounds in dry extracts was expressed as mg GAE per g of dry extract (mg GAE/g DE). All experiments were performed in three replicates, and results are expressed as mean values.

#### 2.9.2. Total Flavonoids Content

The total flavonoids content (TF) was determined in MVPs using aluminum chloride colorimetric assay [21]. Catechin was used as a standard for creation of calibration curve, and absorbance was measured at 510 nm. Content of flavonoids in dry extracts was expressed as mg CE per g of dry extract (mg CE/g DE). All experiments were performed in triplicate, and results were expressed as mean values.

#### 2.9.3. DPPH Assay

The free radical scavenging activity of extracts produced from horehound herbal powder was determined using a simple and fast spectrophotometric method [22]. Briefly, the subcritical extracts were mixed with 90 μM 2,2-diphenyl-1-picryl-hydrazyl (DPPH) and methanol (95%) to provide different final concentrations of extract. After 1 h at room temperature, the absorbance was measured at 517 nm, in triplicates by a 6300 Spectrophotometer (Jenway). Radical scavenging capacity (RSC (%)) was calculated according to Equation (1) and expressed as IC_50_ value, which represents the concentration of extract solution required for obtaining 50% of radical scavenging capacity:

%RSC = 100 − (*A*_sample_ × 100)/*A*_blank_ (1)

where *A*_sample_ is the absorbance of sample solution and *A*_blank_ is the absorbance of control.

#### 2.9.4. FRAP Assay

The reducing power of horehound herbal powder was determined by a previously described method [23]. Various concentrations of subcritical extracts were mixed with sodium phosphate buffer (2.5 mL, 0.2M, pH 6.6) and 2.5 mL of 1% potassium ferricyanide (K_3_Fe(CN)_6_). The mixture was incubated at 50 °C for 20 min. After incubation, 10% trichloroacetic acid aqueous solution (2.5 mL) was added to the mixture, and the mixture was centrifuged for 10 min at 3000 rpm. The obtained supernatant (2.5 mL) was mixed with bidestillated water (2.5 mL) and 0.1% FeCl_3_ solution (0.5 mL). Absorbance was measured at 700 nm. Antioxidant activity was expressed as EC50 value (mg/mL), which causes reduction of 50% Fe^3+^ ions in reaction mixture. All experiments were performed in triplicate.

#### 2.9.5. HPLC Analysis

Phenolic compounds inMVPs samples (MVP 0% MD and MVP 10% MD) were analysed using an Agilent 1200 Series HPLC equipped with a DAD detector (Agilent Technologies, Palo Alto, CA, USA) equipped with Lichrospher^®^ 100 RP 18e column (5 μm, 250 × 4 mm). Mobile phase A was formic acid in water (0.17%), while mobile phase B was acetonitrile. The injection volume was 10 μL, and flow rate 0.8 mL/min with gradient program (0–53 min 0–100% B). Stop time of the analysis was 55 min. Compounds were determined by comparing the retention times and absorption spectra (200–400 nm) of unknown peaks with the reference standards (ferulic acid, *p*-coumaric acid, caffeic acid, rutin, hyperoside, 5-hydroxy-2-methylfurfural). The powders were reconstituted in methanol (1:10), macerated for 24 h and filtered prior to analysis. The investigated samples were analyzed in triplicate.

## 3. Results

### 3.1. Process Efficiency

The optimal spray drying conditions must be satisfied in order to obtain an adequate process efficiency. The dominant factors in spray drying that need to be optimized and monitored throughout the process are feed temperature and air inlet/outlet temperatures [24]. There are several processing obstacles which indirectly affects the properties and yield of the final product. One of them is certainly wall deposition. The wall deposition is created when particles deposit on the surfaces of the inner walls of drying chamber This phenomenon deteriorates the yield of the powder and therefore increase the costs of manufacturing and maintenance [25]. Particles deposit on the wall by attaching to it due to their stickiness which occur above the glass transition temperature, *T*_g_ [26,27]. Apart from monitoring the air inlet temperature, so that on the surface of the product it does not reach more than 10–20 °C above *T*_g_, feed flow rate needs to be constant. When the feed flow rate increases, larger droplets are created and the evaporation rate is lower [28]. When atomizer is supplied with more feed, the particles retain shorter in drying chamber hence the drying time is reduced, contributing in wetter particles. Under these conditions, the particles are more cohesive which cause increase of deposition rate and decrease of yield [25].

Water and ethanol are the most acceptable “green” co-solvents for food-grade products [29]. Despite being safe for human consumption, ethanol has the drawback of being highly flammable, which may limit its wider use in industry. On the contrary, water has the benefits of being nonflammable, flavorless and less restricted in terms of residual solvent. Consequently, the use of water as a common entrainer in a high-pressure extraction process is very attractive and convenient [30]. In our study we used subcritical water extract as liquid feed. The main idea behind introducing subcritical water extraction was to improve extraction yields of desired bioactives and overcome common drawbacks of standard solid-liquid extraction. SWE stands out as a promising technique regarding facilitated analyte diffusion, favoured mass-transfer kinetics, decreased viscosity and surface tension of water when temperature is increased. Temperature is the priority factor that affects efficiency and selectivity of SWE [31]. The water in a subcritical state is used as extraction solvent in SWE. Water is regarded as subcritical at temperatures between 100 °C and 374 °C and at a pressure high enough to keep it in a liquid state [32]. The drying in pilot scale spray-dryers is considered efficient when recovery in the cyclone is higher than 50% [33]. The efficiency of two investigated spray drying processes can be considered high since in both cases it was above 50% (0% MD: η = 58.36%; 10% MD: η = 77.07%). Furthermore, process efficiency was increased by maltodextrin supplementation which can be related to the influence of MD concentration on the formation of surface core prior to the formation of crust enclosing the drying droplets [34]. Finally, regarding all criteria, in the first place absence of stickiness, absence of wall deposition phenomenon and recovery greater than 50%, process conditions of MVPs production can be considered as suitable.

### 3.2. Evaluation of Micrometric Properties and Structure of the Mvps

According to the literature, the diameter of spray-dried particles depends on the several factors including atomization method used, concentration and viscosity of the encapsulated material and finally drying conditions [35]. Some authors have also emphasized that the particle size is significantly affected by the type of carrier, with the largest sizes resulting from using starch or gum arabic as carriers. There are studies that correlated larger particles with an increased encapsulation efficiency [36]. In Table 2 average length, width, perimeter, area and roundness are presented.

**Figure 2:**
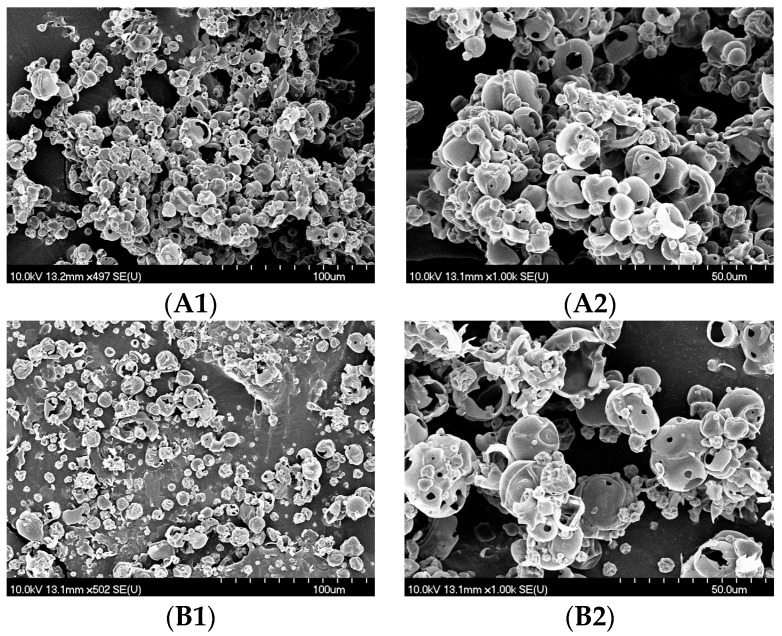
Micrographs of the MVPs particles using SEM with 500× magnification (**A1**, **B1**) and 1000× magnification (**A2**, **B2**) where A1, A2 represent MVP 0% MD and B1, B2 represent MVP 10% MD.

In our study, the existence of maltodextrin caused an increase in the average particle size. In sample MVP 0% MD, particles are smaller (Table 2) than in powder with carrier but aggregation occurred due to presence of cohesiveness. In sample MVP 10% MD, particles are bigger and more scattered which results in lower level of cohesiveness and their appearance as separated, more individual particles (Figure 2).

Figure 2 presents the morphology of the MVPs particles, changed after the SD process, using SEM with 500× (A1, B1) and 1000× (A2, B2) magnifications. As stated in another study [14], before the SD process the raw MD particles were large sized crystals with irregular needle shape. After the SD process, small individual spherical particles with a smooth surface emerged. In our study, particles in both MVPs are nearly spherical with smooth surface. At 1000x magnification, small holes on the particles surface could be detected due to evaporation of solvent. To visually compare A1, A2 with B1, B2 in the SEM pictures, it could be seen that without MD (A1, A2) aggregated postures of particles were produced, however using 10 % MD (B1, B2) the individuality of the particles was determinative. There is a strong adherence of smaller particles to the surface of higher magnitude particles (Figure 2B2) which confirmed the lack of crystalline and the presence of amorphous surfaces.

A decreasing particle size trend when 5% and 10% MD were added was reported [14], while in our study opposite was noticed when 10% MD was added. Tonon et al. also found that a higher maltodextrin concentration in feed solution could lead to the production of larger particles in spray drying, which may be related to the increased feed viscosity with maltodextrin addition [37]. According to Phisut et al., the mean droplet size alters directly with the feed viscosity at constant atomizer speed. The higher the feed viscosity, the larger the droplets created during atomization. Therefore, the larger particles obtained by spray drying [36]. Table 3 lists the particle size distribution of two samples, MVP 0% MD and MVP 10% MD.

**Figure 3:**
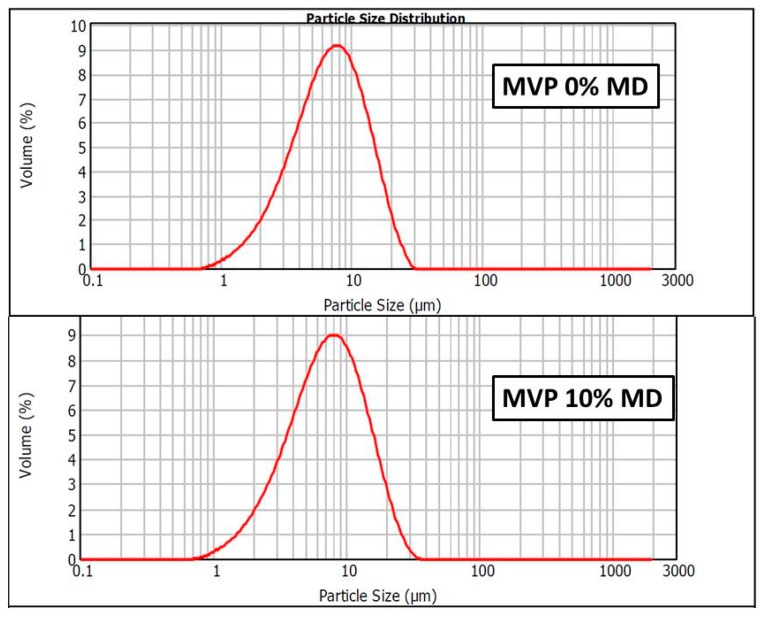
Size distribution of the 0% MD MVP and 10% MD MVP.

Spray-drying of the subcritical extract resulted in microsized particles in both samples, with quite similar distribution (Figure 3). Both distribution curves showed log normal shape. Fine decrease in specific surface area, when 10% MD was added, confirmed that particles are bigger in MVP 10% MD.

**Figure 4:**
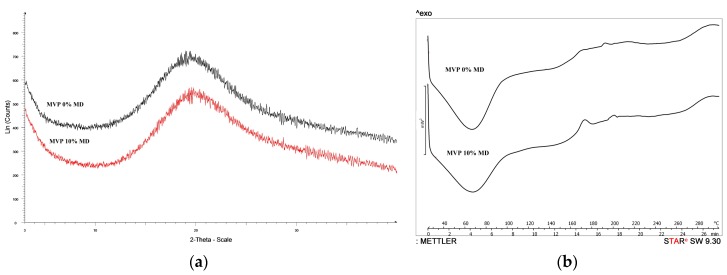
(**a**) XRPD patterns of the 0% MD MVP and 10% MD MVP; (**b**) DSC curves of the 0% MD MVP and 10% MD MVP.

In our previous work [14], the XRPD patterns of untreated and spray-dried MD could be seen, confirming its amorphous character. The results of XRPD analysis (Figure 4a) indicate the amorphous state of analysed MVPs without characteristic peak intensities. The presence of MD did not affect the structure of the MVP extract. The amorphous state is convenient since it can provide very fast dissolution of herbal powders, which is important, e.g., for instant products. The XRPD was confirmed by the thermal behaviors of MVPs. According to DSC curves (Figure 4b), the loss of free water was detected below 100 °C in both cases.

In the case of MVP 10% MD sample, this water content should be bigger, because wider signal could be detected. On the DSC curves, no sharp endotherm peaks were detected which indicates an amorphous character (without melting point of crystalline materials) of both samples.

According to the literature (https://link.springer.com/article/10.1007/s10973-019-08174-z) there could be a *T*_g_ of maltodextrin around 132–150 °C and possibly after its glass transition a small recrystallization could be reached with exothermal event at around 170 °C. During our previous measurements [14], MD decomposed after 300 °C. The solid-phase extract was also amorphous and after its glass transition the recrystallization could be also detected. The structural characterizations were repeated after 6 months (Figure S1) and the character of the amorphous structure was unchanged, which confirmed its stability. The count numbers did not change, which reflected the unaltered amorphous form (500–700 Lin counts).

### 3.3. Moisture Content and Hygroscopicity

The stability, particle size, morphology and rheological behaviour of powders are the main properties affected by moisture content [38]. The lowest moisture content that can be accomplished is favored in terms of adequate storage and manipulation. The most important shift occurs at the glass transition temperature (*T*_g_), which involves a second-order transition from a rubber-like liquid to a glassy solid state [39]. The main consequences of glass transition are the exponential decrease of molecular mobility and free volume, and an increase in viscosity at temperatures below *T*_g_, resulting in structural transformations that are time dependent [40]. Since water has a very low *T*_g_ (−135 °C), it is the major component responsible for the significant *T*_g_ depression observed in food materials. Accordingly, water is considered to be a strong plasticizer in food systems [41] and that is why, if present in high amounts in produced dry powders, water could jeopardize powders quality by decrease of free flowing properties and increase of caking property.

Moisture contents in MVP 0% MD were 4.41% and 3.29% in MVP 10% MD. According to the Ph. Eur. classification method regarding weight gain due to moisture sorption, obtained MVPs can be considered as moderately hygroscopic (2–15% *w*/*w*). The slight decrease in moisture content, with maltodextrin supplemented, was expected. The moisture contents of two obtained horehound powders were similar and lower than 5%, as in the case of *S. montana* powders. This low moisture content can provide sufficient shelf life of the dry extracts due to rare occurrence of microbiological contaminations [42]. Results in the same order of magnitude (3–5%) were previously observed [43] when moisture content of instant tea powder was evaluated. The moisture content of *A. millefollium* powders (6.10–7.68%) showed to be higher than in horehound powders [14]. Our literature review supports the hypothesis that there is an effect of moisture content on the physico-mechanical properties of powders. In pharmaceutical industry, microcrystalline cellulose is a common tableting excipient. The moisture content of microcrystalline cellulose is about 3 to 4%, which is in accordance with the United States Pharmacopeia monograph specifications which restrict the moisture content to no more than 5%. According to these data it is clear that MVPs are adequate not only for application in various food and dietary supplements, but also in the pharmaceutical industry.

Hygroscopicity is also a key property which represents the ability of powder to absorb the moisture from a high relative humidity environment [44]. Hygroscopicities of MVPs were similar, with no significant difference. After 48 h, the hygroscopicity of the investigated carrier-free powder was 21.12% and 19.83% for 10% MD powder. A slight decrease in hygroscopicity was noticed with 10% MD supplementation, which is expected and consistent with moisture content, since MD increases the *T*_g_ of liquid feed. They also observed that the lowest level of hygroscopicity was achieved when the highest maltodextrin concentrations were used [45]. Investigated powder properties are summarized in Table 4.

### 3.4. Water Solubility (WSI) and Water Absorbtion (WAI) Indexes

The wettability is defined as the ability of a powder bulk to be penetrated by a liquid due to capillary forces [46]. The process of dispersing a dry powder into a liquid can be classified into four steps: wetting, submerging, dispersing and dissolving. The physical properties of a powder related with these four steps are usually labeled under the term “instant properties” [47]. The water solubility index (WSI) is an unavoidable parameter in characterization of dry powders since it demonstrates the powders ability to dissolve in water. Opposite to WSI, water absorption index (WAI) shows powder ability to absorb water. High values of WSI and low values of WAI are favourable. In investigated MVPs, WSI were similar and quite high (above 90%) (Table 4). This outcome is expected since liquid feeds were prepared from subcritical extracts where water was used as extractant. WSI slightly decreased as 10% MD was added. The concentration of MD affects the size of the powdered particles and eventually decreases the solubility of the horehound powder. The highest reported WSI for *S. montana* powder with 50% MD was 90.55%. In our study, WAI had preferred low values (WAI = 0.0180 g/g of dry powder for carrier-free sample and WAI = 0.0197 g/g of dry powder for 10% MD sample) comparable with ones obtained for *S. montana* powder with 50% MD [42]. In investigated *A. millefolium* carrier-free and 10% MD powders, WSI were above 70% while WAI were below 20% [14].

### 3.5. MVPs Flow Behavior Properties

One of the most important parameters that characterize powders is definitely their bulk density. The powders have to meet bulk density targets to provide consistent weight during packaging [48]. The higher bulk density and lower moisture content in powder bulk are desired properties for packaging and storage [49]. The bulk density of *Amaranthus* powder increased with a higher maltodextrin concentration [50]. There is a correlation between bulk density and particle size. Particles with smaller size reduced the void spaces among them and arranged themselves in closer form. Consequently, the lower particle size led to a higher bulk density [51]. The bulk densities in investigated MVPs were 83.33 mg/mL in carrier-free powder and 86.96 mg/mL in 10% MD powder. The bulk density was slightly increased with carrier supplementation which is in conrast with published results about decreasing of bulk density of pomegranate powders when MD concentration increased [52]. These values are magnitude of order of *S. montana* powder obtained by adding 10% MD (82.4 mg/mL) [42]. The bulk density measured in *A. millefollium* powder with 10% MD was twice lower (41.31 mg/mL) than bulk densities of MVPs [14]. The cohesive powders favor creation of an open structure supported by the interparticle forces. Consequently, the outcome is a relatively low bulk density of powders [53]. In our case, MVP 0% MD showed good, free flow character while MVP 10% MD showed improved cohesive forces between the particles, however we can state that its flowability is passable (Table 5 and Table S1).

### 3.6. Polyphenol Content in MVPs

Polyphenols comprise one of the most diverse groups of secondary plant metabolites, which possess a wide palette of biological activities, among them antioxidant, anti-inflammatory, antibacterial, and antiviral functions stand out as most relevant [54]. In addition, a large pool of preclinical research and epidemiological data confirm that plant polyphenols can decelerate the progression of some cancers, reduce the risks of cardiovascular disease, neurodegenerative diseases, diabetes and osteoporosis [55,56,57]. Since remarkable bioactive potential has been attributed to polyphenolic compounds, it is necessary to determine their content in dry extracts which could be further implemented in various pharmaceutical formulations and dietary supplements.

In comparison with TP values obtained in 10% MD powders of two herbs, *Satureja montana* and *Achillea millefolium*, total phenols in MVPs (TP = 85.20 mg GAE/g DE in 0% MD sample; TP = 72.98 mg GAE/g DE in 10% MD sample) were lower. Consequently, total flavonoids (TF = 31.37 mg CE/g in 0% MD sample; TF = 26.59 mg CE/g in 10% MD sample) were also lower in relation to TF in *S. montana* powder with 10% MD (TF = 118.69 mg CE/g) [14,42]. Total flavonoids in rosemary powder, obtained by spray drying of ethanolic extract, were comparable with TF values of MVPs [58]. The contents of total phenols and flavonoids decreased with the maltodextrin supplement due to dilution of bioactive compounds encapsulated in powder with inert carrier. Other authors investigated the recovery of encapsulated polyphenols from two *Salvia officinalis* powders (carrier-free and 20% MD). The powders were produced by spray drying of subcritical water extracts. They reported slightly higher values for total phenols (TP = 106.26 mg GAE/g for 0% MD sample and TP = 91.35 mg GAE/g for 20% MD sample) and total flavonoids (TF = 58.97 mg CE/g for 0% MD sample and TF = 56.98 mg CE/g for 20% MD sample). However, they also observed that in extracts obtained by SWE using water as extractant, significantly lower selectivity towards polyphenols was demonstrated in relation to aqueous ethanol applied as extractant in other modern extraction techniques [59]. Polyphenol contents and antioxidant activities for two MVPs are presented in Table 6.

In order to identify dominant phenolic compounds in MVPs, HPLC analyses were performed and the results are presented in Table 7. The major compounds are phenolic acids (ferulic acid, *p*-coumaric acid and caffeic acid) and the flavonoids rutin and hyperoside. It could be observed that in all cases, recoveries of both phenolic acids and flavonoids were distinctly higher when maltodextrin was added as carrier (Table 7). In case of rutin, addition of 10%MD resulted in more than 4-fold increase of rutin content. This suggests that MD addition protects bioactives from thermal degradation.

### 3.7. Antioxidant Activity

There is a discrepancy in the concentrations of polyphenols that are effective in vitro and the ones that are measured in vivo, which are often of an order of magnitude lower. The potency of nutraceuticals to prevent diseases depends on retaining the bioavailability of their active ingredients [60]. Some authors have investigated the retention of antioxidant activity of the encapsulated polyphenols of spray-dried grape seeds, apple skins and olive leaves extracts. They concluded that there is a notable retention of antioxidant activity after encapsulation accomplished by spray drying [61]. In order to test if microencapsulation by spray drying might be useful to protect polyphenols of horehound, two in vitro assays, DPPH and reducing power, were employed. Antioxidant activities of MVPs, expressed as IC_50_ (IC_50_ = 20.4 μg/mL 0% MD sample; IC_50_ = 18.8 μg/mL for 10% MD sample) were lower than the antioxidant activities of herbal powders of *A. millefollium* and *S. montana* obtained in our previous studies [14,42]. However, the obtained antioxidant activities for horehound powders were in line with the IC_50_ values ranging from 17.6 to 24.4 μg/mL of spray dried rosemary hydroalcoholic extract [58]. The reducing power of the horehound powders, expressed as EC_50_ values, were 70.8 μg/mL in 0% MD sample and 75.6 μg/mL in 10% MD sample.

## 4. Conclusions

Spray drying is a well-recognized technique for transforming fruit juices into powders but not so common when the liquid feed is a water/hydroalcoholic extract of plant material. The major challenge in spray drying is the creation of a standardized herbal dried extract that has the required content of active compounds. Since herbal extracts contain numerous chemical constituents and are inconsistent in composition, it is particularly difficult to conform them to a standard. However, this study shows that spray drying of a subcritical horehound extract can be used as a method of choice for obtaining high quality powders which kept the amorphous structure constant after 6 months storage time. Furthermore, recoveries of both phenolic acids and flavonoids were distinctly higher when 10% maltodextrin was added as carrier, which suggests that maltodextrin addition protects bioactives from thermal degradation. This is particularly emphasized in the case of rutin content which was 4-fold higher when carrier was included. Considering the antiasthmatic activity of rutin, this study could initiate developing of a dry powder inhalation formulation based on *M. vulgare* to treat respiratory disorders.

## Figures and Tables

**Table 1:** Composition of prepared formulations prior to spray drying.

Sample	Total Solids [mg/mL]	Amount of Added Maltodextrin [g]	Volume of Spray Dried Liquid Feed [L]	*T*_inlet_ [°C]	*T*_outlet_ [°C]
MVP 0% MD	40.90	0	2	130 ± 5	75–80
MVP 10% MD	44.99	8.18	2	130 ± 5	75–80

**Table 2:** Particle size analyses of MVPs obtained by optical microscope.

Sample	Average Value	Length [μm]	Width [μm]	Perimeter [μm]	Area [μm^2^]	Roundness
MVP 0% MD	Average	4.43	3.57	15.49	14.40	1.33
	SD±	0.12	0.38	0.99	1.17	0.07
MVP 10% MD	Average	6.94	4.37	21.60	23.70	1.55
	SD±	2.65	1.60	6.99	12.92	0.44

**Table 3:** Particle size distribution of MVPs obtained by laser diffraction.

Sample	D 0.1 [μm]	D 0.5 [μm]	D 0.9 [μm]	SSA
MVP 0% MD	2.700	6.920	14.840	1.150
MVP 10% MD	2.791	7.252	15.882	1.100

**Table 4:** Characterization of MVPs obtained from subcritical liquid feed.

Powder Properties	MVP 0% MD	MVP 10% MD
Moisture content (%)	4.41	3.29
Hygroscopicity after 48 h (%)	21.12	19.83
WSI (%)	93.18	91.19
WAI (%)	1.80	1.97

**Table 5:** Flowability expressed by the Carr index and Hausner ratio.

Sample	Carr Index (%)	Hausner Ratio	Flow Character
MVP 0% MD	15.01	1.18	Good/free flow
MVP 10% MD	23.23	1.30	Passable/cohesive

**Table 6:** Content (total phenols (TP) and total flavonoids (TF)) and antioxidant activity of MVPs determined by DPPH and reducing power assays.

Sample	Total Solids [mg/mL]	TP [mg GAE/g]	TF [mg CE/g]	IC_50_ [mg/mL]	EC_50_ [mg/mL]
MVP 0% MD	43.7	85.1975	31.3668	0.0204	0.0708
MVP 10% MD	52.8	72.9810	26.5851	0.0188	0.0756

**Table 7:** Polyphenol content (μg/mL extract) in MVPs obtained using HPLC-DAD.

Sample	Ferulic Acid	*p*-Coumaric Acid	Caffeic Acid	Rutin	Hyperoside
0% MD MVP	48.77	26.42	14.27	134.46	17.43
10% MD MVP	70.69	49.61	20.96	584.55	33.28

## Supplementary Materials

The following are available online at https://www.mdpi.com/1999-4923/11/10/523/s1, Figure S1: XRPD patterns of the MVP 0% MD and MVP 10% MD obtained after 6 months storage time, Table S1: Flow character of powder expressed by Hausner ratio and Carr index.
