# Supplementary material for: Spray Drying of a Subcritical Extract Using Marrubium vulgare as a Method of Choice for Obtaining High Quality Powder
Source: Pharmaceutics. 2019 Oct 11;11(10):523. doi: 10.3390/pharmaceutics11100523 (PMC6835533; doi:10.3390/pharmaceutics11100523)
Supplement: Supplementary file 1 [file pharmaceutics-11-00523-s001.pdf]

pharmaceutics

Supplementary Materials: Spray Drying of a Subcritical Extract Using *Marrubium Vulgare* as a Method of Choice for Obtaining High Quality Powder

Aleksandra Gavarić, Jelena Vladić, Rita Ambrus, Stela Jokić, Piroska Szabó-Révész, Milan Tomić, Marijana Blažić and Senka Vidović

Table 1. Flow character of powder expressed by Hausner ratio and Carr index.

Hausner Ratio	Flow Character	CI (%)
1.00–1.11	Excellent/very free flow	≤10
1.12–1.18	Good/free flow	11–15
1.19–1.25	Fair	16–20
1.26–1.34	Passable	21–25
1.35–1.45	Poor/cohesive	26–31
1.46–1.59	Very poor/very cohesive	32–37
>1.60	Extremely poor/approx. non-flow	>38

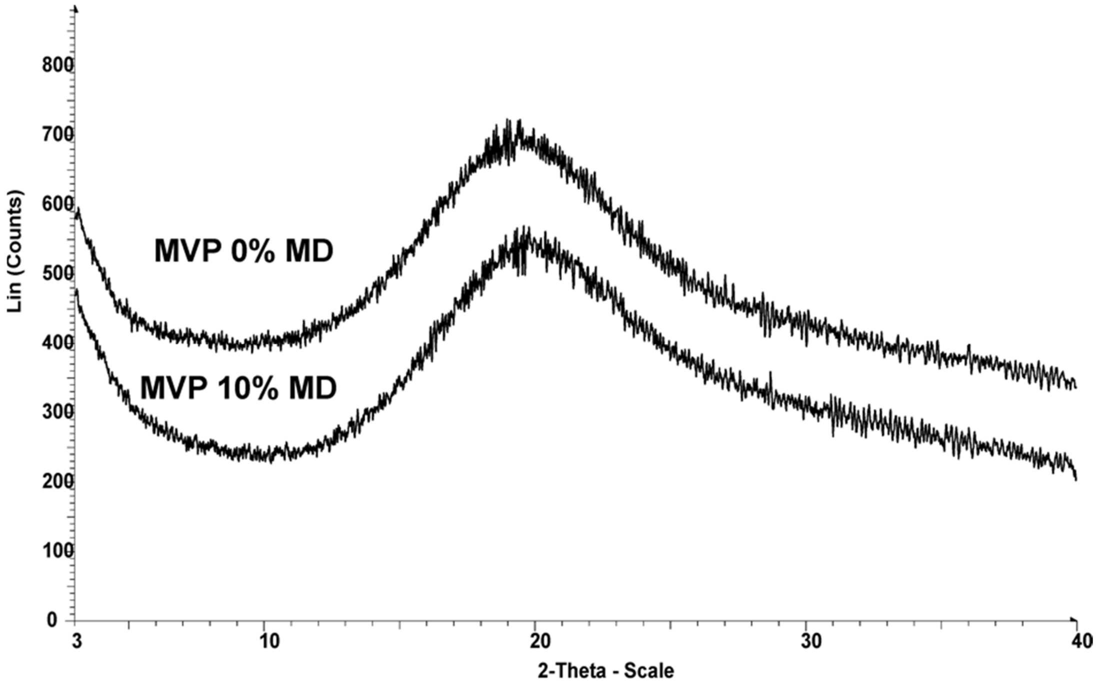

Figure S1. XRPD patterns of the MVP 0% MD and MVP 10% MD obtained after 6 months storage time
